# VITTA TRIAL – safety and performance at 3-year follow-up after implantation of the VIVERE® aortic bioprosthesis

**DOI:** 10.3389/fcvm.2026.1765211

**Published:** 2026-02-18

**Authors:** Luiz Carlos Bettiati Junior, Davi Jean Buoro Cassano, Caio Eduardo Gullo, João Carlos Ferreira Leal

**Affiliations:** 1 Hospital De Caridade São Vicente De Paulo, Jundiaí, Brazil; 2 Hospital Nossa Senhora (Fundação Pio XII), Barretos, Brazil; 3 Hospital Beneficência Portuguesa, São José do Rio Preto, Brazil

**Keywords:** anticalcificant treatment, aortic valve, bioprosthesis, bovine pericardium, cardiac surgery, cardiac valve prosthesis

## Abstract

**Introduction:**

The durability of aortic bioprostheses remains limited by progressive tissue calcification, the main mechanism underlying structural valve deterioration. Aiming to mitigate this process, the VIVERE® Biological Heart Valve Prosthesis, treated with the REALOG® anticalcification technology, was developed to reduce degenerative effects associated with glutaraldehyde fixation.

**Objective:**

To evaluate the safety, clinical performance, and hemodynamic outcomes of the VIVERE® Biological Heart Valve Prosthesis over 36 months following aortic valve replacement.

**Methods:**

Retrospective, multicenter, single-arm study included patients with aortic valve disease who underwent valve replacement using VIVERE® bioprosthesis. Clinical success (valve implantation without complications and no major adverse event until hospital discharge), linearized rates of major adverse events—composite and valve-related (death and/or stroke and/or reintervention), survival, clinical efficacy (NYHA class I–II), and hemodynamic performance (valve area, mean gradient, and presence of regurgitation or paravalvular leak) were assessed. Follow-up was conducted for up to 36 months after implantation. Continuous variables were expressed as mean ± standard deviation, and categorical variables as frequencies and percentages. Survival was assessed by Kaplan–Meier analysis, and linearized event rates expressed per 100 patient-years.

**Results:**

106 patients were included, with a mean age of 67 ± 11.6 years. Clinical success was observed in 88.7% of patients, with a linearized rate of valve-related major adverse composite events of 1.0% per patient-year and a 36-month survival of 87.7%. At 36 months, there was an 80% reduction in mean gradient and a 71% increase in effective orifice area, with 87.5% of patients in NYHA functional class I–II. No structural valve deterioration or paravalvular leak was observed at 36 months.

**Conclusion:**

At 36 months, VIVERE® demonstrated favorable valve-related safety and efficacy, with low valve-related mortality, stable hemodynamics, and sustained functional improvement.

## Introduction

Aortic valve diseases have increased over the years, particularly aortic valve stenosis (1, 2). These conditions significantly affect patient prognosis and quality of life (3). In patients younger than 70 years with low or intermediate surgical risk and/or those requiring concomitant procedures, surgical aortic valve replacement remains the recommended treatment (2, 4). Over the past decades, bioprostheses made from bovine pericardium have been widely used due to their favorable hemodynamic performance and the advantage of not requiring lifelong anticoagulation, unlike mechanical valves (5). However, calcification remains the main factor limiting bioprosthesis durability (6).

Bioprosthetic calcification is a multifactorial process resulting from both material-related characteristics and the host's biological response. Calcium deposition occurs primarily in devitalized cells and cellular debris remaining after glutaraldehyde fixation, which serve as nucleation sites for mineral precipitation (7, 8). Additional factors—such as mechanical stress, inflammation, immune response to glycans (α-Gal, Neu5Gc), thrombosis, and proteolytic degradation—accelerate degenerative processes, especially in younger patients or those with comorbidities such as renal insufficiency and diabetes (9, 10).

In light of these limitations, several anticalcification strategies have been developed to extend bioprosthesis durability. Among these, the VIVERE® bioprosthesis incorporates a tissue-processing approach specifically designed to mitigate calcification (11).

The VIVERE® bioprosthesis, a second-generation derivative of the Bovine Pericardium Organic Valvular Bioprosthesis (BVP), Braile, São José do Rio Preto, Brazil, incorporates the REALOG® anticalcification treatment as its main innovation. This process aims to block residual aldehyde groups and promote oxidation of glutaraldehyde-induced crosslinks, thereby reducing calcification and cytotoxicity without compromising the mechanical properties of the tissue (12, 13).

Although several studies have demonstrated the safety and efficacy of the BVP (14, 15), robust evidence regarding the medium-term clinical and hemodynamic outcomes of the VIVERE® bioprosthesis is still lacking. Therefore, the aim of this study is to analyze these outcomes over a 36-month follow-up period.

## Methods

This retrospective, multicenter, single-arm study included patients who underwent surgical aortic valve replacement between 2017 and 2022. Data were obtained through medical record review from three specialized cardiology hospitals in Brazil (Hospital de Caridade São Vicente de Paulo – Jundiaí; Hospital Nossa Senhora – Fundação Pio XII de Barretos; Hospital Beneficência Portuguesa – São José do Rio Preto).

Data were collected at preoperative/baseline, during the procedure, at hospital discharge, and during follow-up at 30 days, 6 months, and annually up to 36 months. At preoperative/baseline, demographic characteristics (age, sex, and body mass index), clinical and functional status (EuroSCORE II and NYHA class), etiology of valvular disease, prior cardiovascular interventions, and echocardiographic parameters, including left ventricular ejection fraction (LVEF), mean aortic gradient, and effective orifice area, were recorded. Procedural data included the type of surgical procedure, size of the implanted valve, cardiopulmonary bypass and aortic cross-clamp times, echocardiographic parameters, and perioperative adverse events. Early safety outcomes, defined as clinical success (valve implantation without complications or major adverse events), were assessed at hospital discharge. During follow-up, late safety and performance outcomes were assessed. Late safety outcomes included the linearized rate of valve-related major adverse events (death, stroke, and/or reintervention) and 36-month survival. Performance outcomes comprised clinical efficacy, defined as the proportion of patients in NYHA class I–II, and hemodynamic performance, measured by valve area (cm^2^), mean gradient (mmHg), and presence of regurgitation or paravalvular leak (absent/trivial, mild, moderate, or severe). Linearized event rates were calculated per patient, and follow-up performance outcomes were compared with baseline values (16, 17).

All patients provided informed consent, and the ethics committees of the participating institutions approved the study protocol. Analyses were conducted using anonymized data, and the study was registered at ClinicalTrials.gov (NCT06959836).

Continuous variables were expressed as mean ± standard deviation, and categorical variables as frequencies and percentages. Survival was assessed using the Kaplan–Meier method, and linearized event rates were expressed per 100 patient-years. For patients without available clinical follow-up, survival was verified through the Brazilian National Mortality Registry. Patients confirmed to be alive were included in the calculation of patient-years.

Statistical significance was defined as *p* < 0.05 and analyses were performed using Stata 15.1® (StataCorp, LLC, College Station, TX, USA).

### Investigational device

**Figure 1:**
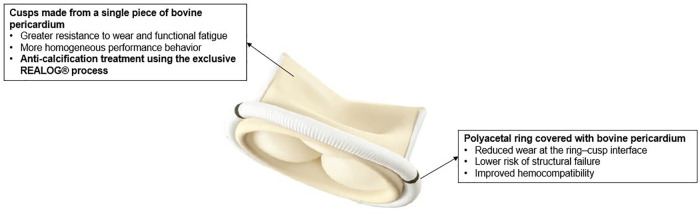
Structural design and material composition of the bovine pericardial biologic prosthetic valve VIVERE®.

Like the BVP, the VIVERE® is a bioprosthesis constructed with a coated polyacetal ring and three leaflets obtained from a single strip of bovine pericardium (Figure 1).

**Figure 2:**
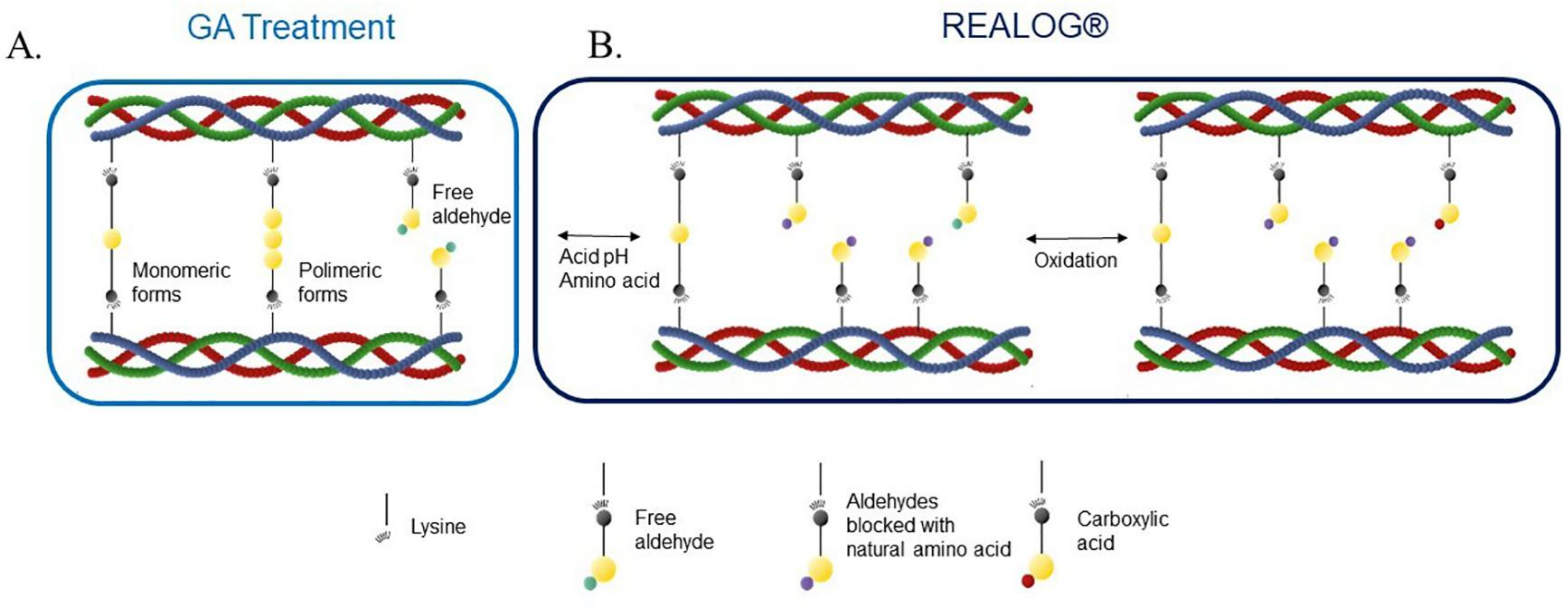
Schematic representation of the REALOG® anticalcification treatment mechanism. **(A)** GA fixation stabilizes the collagen matrix through cross-linking, but leaves residual free aldehyde groups in monomeric and polymeric forms, primarily associated with lysine residues (free aldehydes are known nucleation sites for calcium deposition). **(B)** The REALOG® process involves the use of amino acids and an oxidation step. It chemically blocks free residual aldehyde groups through reactions with amino acids under acidic conditions, followed by an oxidation step that converts any remaining aldehyde groups into carboxylic acids. This process reduces calcium affinity, thereby mitigating tissue calcification while preserving the structural integrity of the collagen matrix. GA, glutaraldehyde.

However, the VIVERE® differs from the BVP in its ability to be implanted either intra- or supra-annularly and in its use of the REALOG® treatment, which reduces calcification and enhances the biocompatibility of the bioprosthesis. Glutaraldehyde treatment, as used in the BVP stabilizes collagen through the formation of crosslinks between lysine amine groups, both via monomeric and polymeric reactions, creating a network that increases mechanical strength and the durability of the biological tissue. Nevertheless, this process may leave residual free aldehydes, which are associated with cytotoxicity and calcification. To minimize these residual aldehydes, the VIVERE® is treated with REALOG® technology, which involves processing with the natural amino acid (glutamic acid) to stabilize the matrix, followed by an oxidative step that converts free aldehydes into carboxylic acids with lower chemical reactivity, thereby improving pericardial biocompatibility (12). Thereafter, the VIVERE® bioprosthesis is stored in an aldehyde-based liquid preserving solution (11). Figure 2 illustrates the glutaraldehyde (GA) treatment and the chemical processes involved in the REALOG® treatment applied to bovine pericardial tissue following GA fixation.

**Figure 3:**
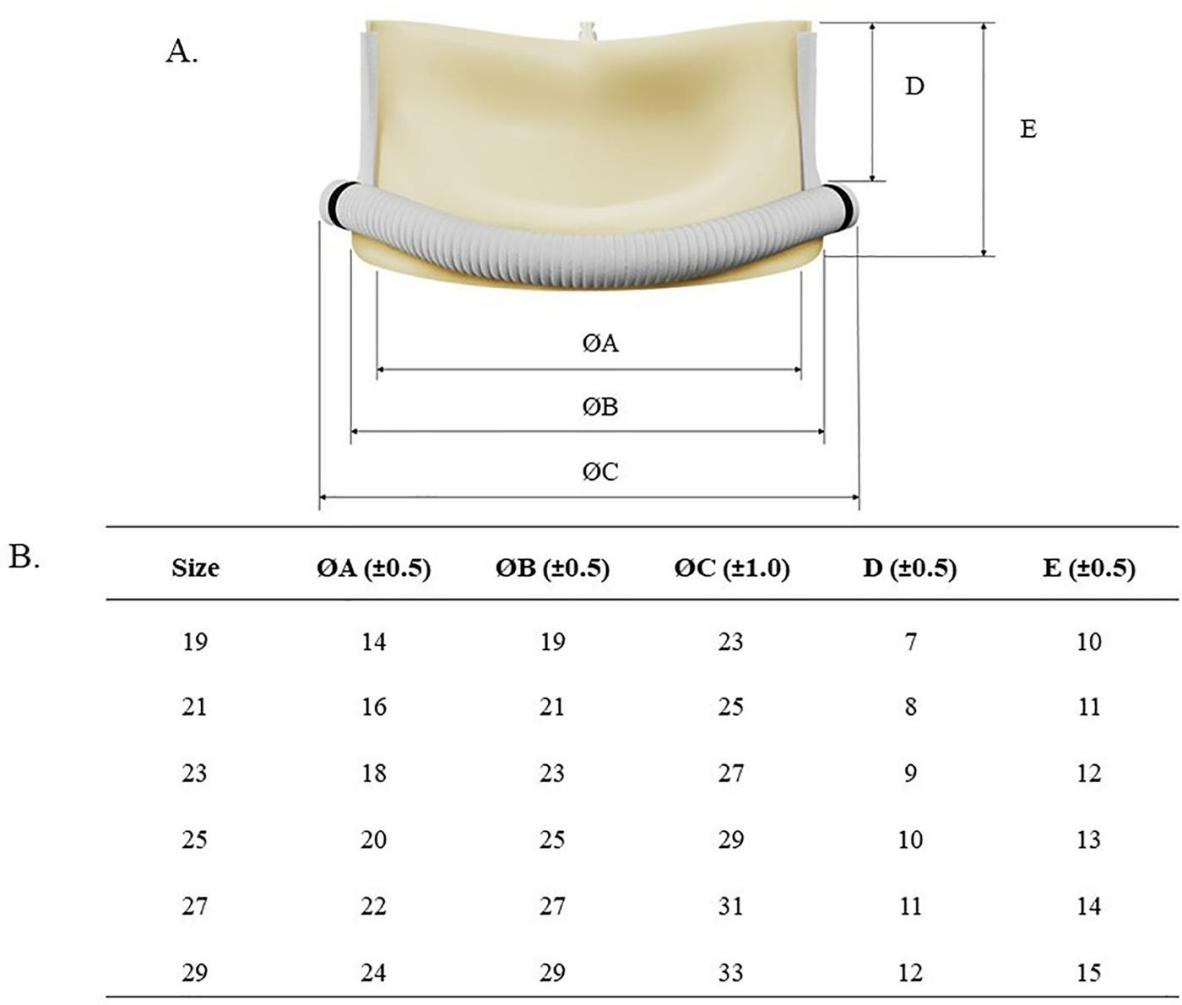
Dimensional characteristics and size specifications of the VIVERE® bioprosthesis. **(A)** Schematic representation illustrating the definition of the main dimensional parameters: internal diameter (ØA), external diameter (ØB), external diameter of the suture ring (ØC), rod height **(D)**, and overall height **(E) (B)** Corresponding dimensional values for each valve size. All measurements are reported in millimeters.

Similar to the BVP, the VIVERE® is available in a wide range of sizes for the aortic position, designed to meet the various anatomical needs of patients undergoing valve replacement. The available VIVERE® sizes for the aortic position are 19 mm, 21 mm, 23 mm, 25 mm, 27 mm, and 29 mm (Figure 3). These size options also allow cardiac surgeons to select the most appropriate valve for each patient, ensuring optimal functionality and compatibility after surgery.

## Results

A total of 106 patients, aged 30–83 years, were included across the three participating centers (Supplementary Figure S1), with 65.1% being male. The primary indication for valve implantation was aortic stenosis of calcific degenerative etiology (41.5%). Mean aortic gradient and effective orifice area values reflected severe aortic stenosis, whereas left ventricular ejection fraction indicated preserved ventricular function. Baseline characteristics of the patients undergoing aortic valve implantation are summarized in Table 1.

**Table 1:** Baseline characteristics of the patients.

Variables	Mean ± SD or *n* (%)^a^
Demographic characteristics	
Age (years)	67 ± 11.6
BMI (Kg/m^2^)	27.8 ± 4.6
Male sex	69 (65.1)
Clinical and functional assessment	
EuroScore II	2.0 ± 4.6
NYHA, *n* (%)^b^	
I	6 (5.7)
II	45 (42.4)
III	16 (15.1)
IV	27 (25.5)
Etiology of valvular disease	
Calcification	44 (41.5)
Congenital	9 (8.5)
Rheumatic	9 (8.5)
Infectious	13 (12.3)
Previous prosthesis degeneration	8 (7.5)
Other	23 (21.6)
Previous cardiovascular interventions	
Myocardial revascularization	13 (12.3)
Permanent pacemaker implantation	1 (0.9)
Surgical aortic valve replacement	8 (7.5)
Echocardiographic data^b^	
LVEF (%)	59.7 ± 12.0
Mean gradient (mmHg)	45.0 ± 21.1
Effective orifice area (cm^2^)	0.9 ± 0.5

*n*, number of individuals; SD, standard deviation; BMI, body mass index; EuroSCORE, European system for cardiac surgery risk assessment; NYHA, New York Heart Association; LVEF, left ventricular ejection fraction; Mean gradient, transvalvular mean gradient.

^a^ Continuous variables are presented as mean ± SD, and categorical variables as number (%).

^b^ Data not available for all participants.

Isolated valve replacement was the most frequent procedure, occurring in 62 of 106 patients (58.5%), followed by valve replacement with concomitant aortic interventions in 18 patients (17.0%), valve replacement with coronary artery bypass grafting in 15 patients (14.1%), and double valve replacement in 11 patients (10.4%). The mean cardiopulmonary bypass time was 78 ± 24 min (range, 60–285 min), and the mean aortic cross-clamp time was 52 ± 18 min (range, 50–219 min). Valve size distribution was as follows: 19 mm in 3 patients (2.8%), 21 mm in 16 patients (15.1%), 23 mm in 27 patients (25.5%), 25 mm in 36 patients (34.0%), 27 mm in 19 patients (17.9%), and 29 mm in 5 patients (4.7%).

### Safety outcomes

Early safety outcomes, assessed by clinical success, was achieved in 88.7% of patients (94/106). Among the 12 patients in whom clinical success was not achieved, 9 (75%) died, 1 (8.3%) developed sepsis, 1 (8.3%) required reoperation for pericardial drainage, and 1 (8.3%) experienced life-threatening bleeding. Of the 9 deaths, 2 (22.2%) were due to septic shock, 2 (22.2%) to acute renal failure, 1 (11.1%) to stroke, 1 (11.1%) to mixed shock, 1 (11.1%) to pulmonary embolism, and 2 (22.2%) to infective endocarditis. Early valve-related mortality was 2.8% (3/106 patients). Among the patients who died, 5/9 (55.6%) were male, 2/9 (22.2%) had undergone more than one previous valve surgery, and 5/9 (55.6%) underwent multiple valve replacement or another concomitant procedure. These patients also presented longer cardiopulmonary bypass times (mean, 154 min).

**Figure 4:**
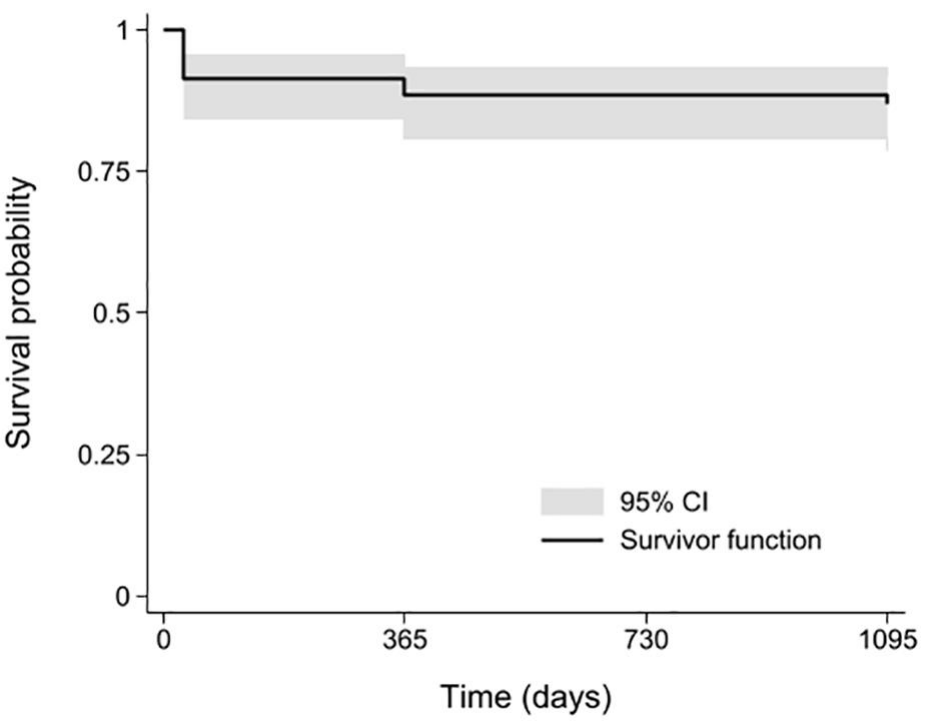
Time course of survival (post-procedure to 36 months).

For the evaluation of late safety outcomes, survival analysis included the 9 early deaths and 4 late deaths unrelated to the device (two deaths due to congestive heart failure at 12 months and two due to malignancy at 12 and 36 months). Accordingly, overall survival at 36 months was 87.7% (95% CI: 82.4%–94.9%), as shown in the Kaplan–Meier curve (Figure 4).

The 36-month freedom from valve-related composite major adverse events was 97.2% (103/106 patients). The linearized rate of valve-related composite major adverse events was 1.0% per patient-year (over 288 patient-years), consisting of three patients who experienced device-related deaths (two due to infective endocarditis and one following stroke). No surgical reinterventions related to the bioprosthesis were required.

Early adverse event rates (non-linearized, based on 106 patients) and late adverse event rates (linearized, based on 288 patient-years) related to valve implantation are summarized in Table 2.

**Table 2:** Valve-related adverse events.

Adverse events	Early adverse events rate^a^ *n* (%)	Linearized late adverse events rate^a^^,^^b^ *n* (%)	Adverse events -free rate (95%CI)^c^
Device-related death	3 (2.8)	3 (1.0)	97.2% (94.1–100)
Stroke	1 (0.9)*	1 (0.3)	99.1% (97.2–100)
Valve thrombosis	–	–	100%
Major bleeding	4 (3.7)	4 (1.4)	96.4% (92.7–99.9)
Endocarditis	2 (1.8)*	2 (0.7)	98.1% (95.6–100)
Moderate or severe paravalvular leak	1 (0.9)	1 (0.3)	99.1% (97.2–100)
Reintervention	–	–	100%
Structural valve deterioration^&^	–	–	100%

CI, confidence interval; Stroke, cerebrovascular accident; *n*, number of cases.

^a^ Events occurring within 30 days after the procedure; calculated as the number of events divided by the number of patients who underwent the procedure.

^b^ Cumulative late events per patient-year, calculated by dividing the number of events by the total patient-years of follow-up. Patient-years = sum of individual follow-up times in years (total: 288 patient-years).

^c^ Event-free rates were calculated based on Kaplan–Meier analysis.

* Patients also experienced device-related death.

^&^ Calcification, fibrosis, and/or leaflet fracture.

### Performance outcomes (late outcome)

**Figure 5:**
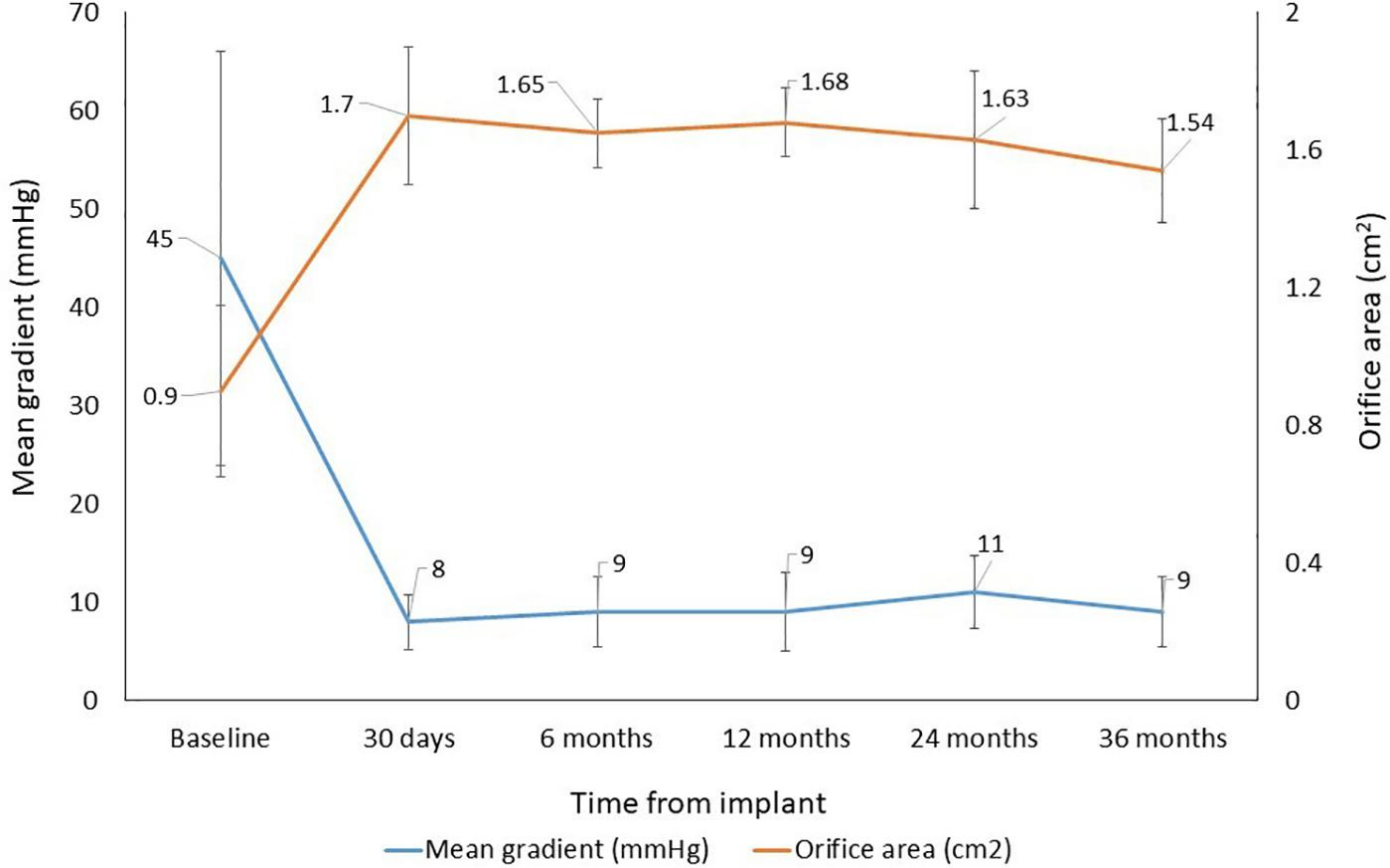
Time course of hemodynamic performance (baseline to 36 months).

In the assessment of hemodynamic performance, a sustained reduction in mean gradient was observed, decreasing from 45 ± 21.1 mmHg at baseline to 9.0 ± 3.6 mmHg at 36 months, corresponding to an 80% reduction (*p* < 0.001) (Figure 5). Effective orifice area increased from 0.9 ± 0.5 cm^2^ at baseline to 1.54 ± 0.4 cm^2^ at 36 months, representing a 71% increase (*p* < 0.001). By valve size, mean gradient at 36 months was slightly higher than that observed at 30-day follow-up for the 21 mm and 29 mm valves (Supplementary Figure S2).

No patient experienced moderate or severe paravalvular leak immediately after valve implantation. At 6 months, 1 of 106 patients (0.9%) had a moderate paravalvular leak, which persisted through 12 months and resulted in death between the 12- and 24-month follow-ups. At 36 months, no paravalvular leaks ≥ moderate were reported.

**Figure 6:**
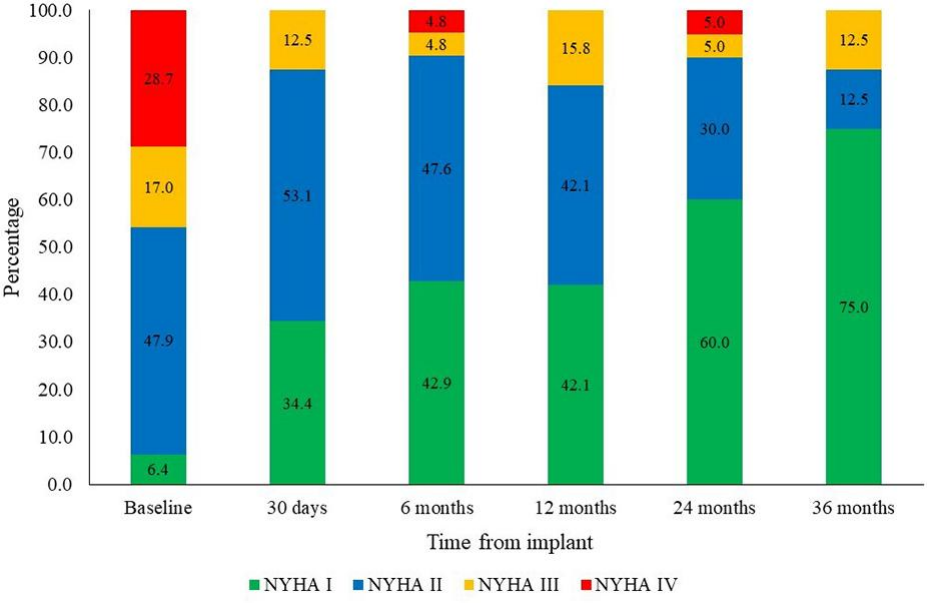
Time course of NYHA functional class (baseline to 36 months).

Regarding clinical efficacy, 87.5% of patients were in NYHA functional class I–II at 36 months. Figure 6 illustrates NYHA functional class over the follow-up periods.

## Discussion

The VIVERE® bioprosthesis demonstrated excellent clinical outcomes at 36 months. Key findings included clinical success in 88.7% of patients, early valve-related mortality of 2.8%, and a linearized rate of valve-related composite major adverse events of 1.0% per patient-year at 36 months. In terms of hemodynamic and clinical performance, mean gradient decreased from 45 to 9 mmHg, effective orifice area increased from 0.9 to 1.54 cm^2^, and 87.5% of patients were in NYHA functional class I–II at 36 months.

The clinical success rate of 88.7% observed with VIVERE® implantation was lower than the rates of 96.3% and 97.3% reported in Japanese patients receiving the Magna Ease and Avalus™ valves, respectively (18). Early valve-related mortality of 2.8% was higher than that reported after Magna Ease (0%) (19) and Dafodil™ (1.5%) implantation, and overall survival was 87.7%, approximately 6% lower than that observed after Dafodil™ implantation (20). The lower clinical success rate was largely driven by in-hospital deaths, mainly due to sepsis, shock, acute renal failure, and endocarditis, likely reflecting structural limitations of healthcare delivery in middle-income countries (21). These limitations include scarce diagnostic resources, delayed diagnosis, restricted availability of supportive therapies, and limited intensive care unit capacity (21, 22), factors that may delay the response to complications (21) and, consequently, contribute to the higher mortality observed in our cohort.

It should be emphasized that all patients in this study were treated within the Brazilian Unified Health System (SUS), in which admission of patients with more severe clinical conditions is more frequent, including higher rates of circulatory shock, renal failure, and in-hospital mortality (23). Moreover, treatment in SUS hospitals has been identified as an independent risk factor for sepsis-related mortality (22). This healthcare context and higher baseline clinical severity have important implications for the accuracy of surgical risk prediction models. Although the cohort presented a low estimated operative risk (mean EuroScore II, 2.0 ± 4.6), EuroScore-based models may underestimate actual mortality in settings such as SUS, particularly in low-risk populations and in middle-income countries, where socioeconomic factors, delayed disease presentation, increased comorbidity burden, and limited access to diagnostic and therapeutic resources are not fully captured by traditional risk stratification tools (24). In this context, nationwide Brazilian data from a cohort of 78,806 patients undergoing heart valve surgery reported an in-hospital mortality rate of 7.6% (25), while multicenter registry data from the BYPASS study demonstrated a similar hospital mortality of approximately 7.3% (26). Although these rates are numerically lower than the overall mortality observed in the present study, they underscore the non-negligible early mortality associated with surgical valve replacement in public healthcare systems.

With regard to endocarditis, one patient with infective endocarditis had pre-existing congenital heart disease, a recognized risk factor for valvular infection (27). Another relevant factor was patient age: nearly 77% (10/13) of patients who died after VIVERE® implantation were older than 60 years. A retrospective study conducted in Russian centers between 2009 and 2018 identified advanced age as the strongest predictor of reduced survival, with mortality rates of 13.7% among patients aged 60–74 years and 24.7% among those aged ≥75 years at 8 years post-implantation (28).

Despite these findings, it is important to emphasize that the low valve-related mortality and the low linearized rate of valve-related major adverse events observed in this study support the procedural safety of the VIVERE® bioprosthesis.

Comparative analysis of clinical and hemodynamic outcomes at 36 months indicates that VIVERE® performance is consistent with that of other commercially available bioprostheses. The mean gradient of 9.0 ± 3.6 mmHg and effective orifice area of 1.54 ± 0.4 cm^2^ at 36 months may be related to the structural characteristics of the valve. The single bovine pericardial patch reduces the number of sutures and may contribute to a larger effective orifice area, favoring lower flow resistance and better hemodynamic adaptation. Moreover, the cylindrical shape, lower height, larger valve area, and supra-annular design are associated with laminar flow, lower pressure gradients, and improved hemodynamic performance (18).

The mean gradient observed throughout follow-up (8–11 mmHg) was lower than that reported for Perimount Magna Ease valves at 2 years (13.3 ± 5.5 mmHg) (19), at 3 years (12 ± 5.1 mmHg) (29), and Avalus™ at 3 years (13.0 ± 5.1 mmHg) (30). The mean effective orifice area (1.8 cm^2^) during follow-up was similar to that reported for Dafodil™ at 3 years (20) and slightly higher than the 1.6 cm^2^ observed for Avalus™ at 2 years (30) and Perimount Magna Ease at 4 years (19). These findings suggest sustained hemodynamic benefit over the long term and are consistent with nearly 90% of patients maintaining NYHA class I–II during 3-year follow-up, in agreement with previous reports (14, 15).

As with Avalus™ (31) and Dafodil™ (20), no structural valve deterioration or need for reintervention was observed at 36 months. These results may be attributed to the REALOG® anticalcification treatment, highlighting the potential of the prosthesis as a long-term option. Furthermore, VIVERE® shares the basic platform of the BVP valve (Braile), which has documented durability exceeding 26 years (14).

The relationship between lower mean gradient and larger effective orifice area is particularly relevant, as these parameters are associated with reduced patient-prosthesis mismatch, a complication that can negatively affect the clinical and structural outcomes of bioprostheses (20). Therefore, the performance of the VIVERE® bovine pericardial bioprosthesis, combined with the REALOG® anticalcification treatment, may represent an advance in valve durability, although extended follow-up studies are required to confirm this hypothesis.

In summary, the results demonstrate functional improvement, absence of structural deterioration, and consistent clinical and hemodynamic performance of the VIVERE® bioprosthesis, with sustained symptomatic relief and functional benefit. However, prolonged follow-up (7–10 years) is needed to confirm the long-term durability of the anticalcification technology.

This study has some limitations. First, the follow-up duration is limited to mid-term outcomes, which precludes definitive conclusions regarding long-term structural valve durability. Second, the variation in the number of patients enrolled per center may have influenced the observed outcomes; however, due to the small sample size in some centers, a center-specific analysis was not performed. Additionally, no clinical event adjudication committee was used, and there was no central echocardiography core laboratory, which may have introduced variability in event assessment and imaging interpretation. Finally, the absence of contemporaneous comparative cohorts with alternative surgical bioprostheses in the participating institutions limits direct benchmarking. Although multiple valve platforms are commercially available, the centers involved in this study did not maintain parallel datasets using other devices, precluding within-institution comparisons. As this was a single-arm study, external reference data were required to contextualize the findings. Therefore, the discussion was based on bioprosthetic valves with comparable design and preservation characteristics—namely, bovine pericardial valves intended for open surgical implantation and stored in liquid-based solutions. Newer-generation bioprostheses incorporating advanced solutions that allow dry storage prior to surgical implantation (32, 33) represent a distinct technological class; thus, any indirect comparison with such platforms would be inherently confounded by differences in tissue preservation.

## Conclusion

At 36 months of follow-up, the VIVERE® bioprosthesis was associated with favorable valve-related safety and efficacy outcomes, including low valve-related mortality, stable hemodynamic performance, and sustained functional improvement.

## Data Availability

The raw data supporting the conclusions of this article will be made available by the authors, without undue reservation.
