# Supplementary material for: VITTA TRIAL – safety and performance at 3-year follow-up after implantation of the VIVERE® aortic bioprosthesis
Source: Front Cardiovasc Med. 2026 Feb 18;13:1765211. doi: 10.3389/fcvm.2026.1765211 (PMC12957083; doi:10.3389/fcvm.2026.1765211)
Supplement: Supplementary file 1 [file Datasheet1.docx]

**Supplementary figure 1.** Inclusions by center, in percentage.

n=number of participants included

**Supplementary figure 2.** Time course of hemodynamic performance, per valve size (30 days to 36 months).

|  | Mean gradiente (mmHg) | | | | |
| --- | --- | --- | --- | --- | --- |
| Valve size | 30 days | 6 months | 12 months | 24 months | 36 months |
| 21 mm | 5 | 7 | 9 | 9 | 9 |
| 23 mm | 7 | 6 | 7 | 7 | 7 |
| 25 mm | 8 | 8.5 | 9.6 | 10 | 6 |
| 27 mm | 14.8 | 11 | 9.5 | 12.3 | 10.3 |
| 29 mm | 6 | 10 | 12 | 12 | 9 |
